# Multilevel Conductance States of Vapor‐Transport‐Deposited Sb_2_S_3_ Memristors Achieved via Electrical and Optical Modulation

**DOI:** 10.1002/advs.202405251

**Published:** 2024-07-03

**Authors:** Somnath S. Kundale, Pravin S. Pawar, Dhananjay D. Kumbhar, I. Ketut Gary Devara, Indu Sharma, Parag R. Patil, Windy Ayu Lestari, Soobin Shim, Jihye Park, Tukaram D. Dongale, Sang Yong Nam, Jaeyeong Heo, Jun Hong Park

**Affiliations:** ^1^ Department of Materials Engineering and Convergence Technology Gyeongsang National University Jinju Gyeongsangnam‐do 52828 Republic of Korea; ^2^ Research Institute for Green Energy Convergence Technology Gyeongsang National University Jinju 52828 Republic of Korea; ^3^ Department of Materials Science and Engineering, and Optoelectronics Convergence Research Center Chonnam National University Gwangju 61186 Republic of Korea; ^4^ Computational Electronics and Nanoscience Research Laboratory, School of Nanoscience and Biotechnology Shivaji University Kolhapur 416004 India

**Keywords:** antimony sulfide, memristors, optical switching, synaptic application, vapor transport deposition

## Abstract

The pursuit of advanced brain‐inspired electronic devices and memory technologies has led to explore novel materials by processing multimodal and multilevel tailored conductive properties as the next generation of semiconductor platforms, due to von Neumann architecture limits. Among such materials, antimony sulfide (Sb_2_S_3_) thin films exhibit outstanding optical and electronic properties, and therefore, they are ideal for applications such as thin‐film solar cells and nonvolatile memory systems. This study investigates the conduction modulation and memory functionalities of Sb_2_S_3_ thin films deposited via the vapor transport deposition technique. Experimental results indicate that the Ag/Sb_2_S_3_/Pt device possesses properties suitable for memory applications, including low operational voltages, robust endurance, and reliable switching behavior. Further, the reproducibility and stability of these properties across different device batches validate the reliability of these devices for practical implementation. Moreover, Sb_2_S_3_‐based memristors exhibit artificial neuroplasticity with prolonged stability, promising considerable advancements in neuromorphic computing. Leveraging the photosensitivity of Sb_2_S_3_ enables the Ag/Sb_2_S_3_/Pt device to exhibit significant low operating potential and conductivity modulation under optical stimulation for memory applications. This research highlights the potential applications of Sb_2_S_3_ in future memory devices and optoelectronics and in shaping electronics with versatility.

## Graphical abstract

The potential of antimony sulfide (Sb_2_S_3_) thin films for cutting‐edge electronic memory technologies is investigated in this work. Incorporates the vapor transport deposited Sb_2_S_3_ thin‐film conduction modulation and memory functions. Ag/Sb_2_S_3_/Pt devices are highly suitable for memory applications because they have low operating voltages, strong durability, artificial neuroplasticity with long‐term stability, and they show promise for future developments in neuromorphic computing. The Ag/Sb_2_S_3_/Pt device exhibits a notable change of conductivity upon optical stimulation, underscoring its potential applications in memory and optoelectronic systems in the future.

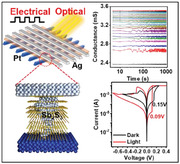

## 1 Introduction

Memristors, a blend of “memory” and “resistor,” can regulate resistance across multiple states, effectively storing the history of past electrical stimuli, and they have emerged as a pivotal electronic component in the field of memory storage devices and brain‐inspired neuromorphic computing.^[^ ^1^ ^]^ Inspired by biological mechanisms, neuromorphic computing designs energy‐efficient artificial neurons and synapses that can emulate brain‐like learning and memory processes for solving complex tasks.^[^ ^2^ ^]^ Memristive artificial synapses can realistically mimic synaptic plasticity and have emerged as fundamental components for neuromorphic systems, offering promising prospects to construct energy‐efficient circuitry and facilitate advanced information processing.^[^ ^1^, ^3^ ^]^ Research on materials for resistive switching (RS) devices has actively explored oxides,^[^ ^4^ ^]^ carbides,^[^ ^5^ ^]^ halides,^[^ ^6^ ^]^ chalcogenides,^[^ ^7^ ^]^ metal‐organic frameworks,^[^ ^8^ ^]^ and graphene‐based composites,^[^ ^9^ ^]^ because of their diverse properties and applications in memory technology and neuromorphic computing. Among these materials, chalcogenide materials have gained significant attention because of their exceptional physical attributes, electrical adjustability, low‐power switching capabilities, and compatibility with hetero‐integration.^[^ ^1^, ^10^ ^]^ Consequently, several experimental studies focused on memristors based on chalcogenide materials or tailored conductive properties, revealing their distinct memristive behaviors and innovative synaptic functionalities, diverging from conventional bulk‐material‐based systems.^[^ ^11^ ^]^ Recent advancements within neuromorphic computing have centered on fabricating memristors with efficiency, reproducibility and low power consumption.^[^ ^12^ ^]^

The exploration of novel chalcogenide materials with tailored conductive properties by electrical and optical inputs has emerged as a critical avenue for advancing electronic devices and memory technologies.^[^ ^13^ ^]^ Among these materials, antimony sulfide (Sb_2_S_3_) thin films have gained significant attention because of their abundance in the Earth's crust, environmentally friendly constituents, and excellent optical and electronic properties, which further contribute to their versatility and result in potential platforms for switching devices with multimodality.^[^ ^14^ ^]^ Sb_2_S_3_, known for its photovoltaic properties, is commonly utilized as a sensitizing and absorbent layer in thin‐film solar cells. Sb_2_S_3_ can convert sunlight into electrical energy efficiently, making it a potential component for enhancing the performance of solar‐cell devices.^[^ ^15^ ^]^ In addition, Sb_2_S_3_ has a bandgap ranging from 1.5–1.8 eV, along with a high optical absorption coefficient in the visible ray range.^[^ ^16^ ^]^ The main advantage of the Sb_2_S_3_ is their optimal band gap and high absorption coefficient compared to oxides (TiO_2_, CuO_x_, HfO_2_) and chalcogenides (MoS_2_, WS_2_, NiS_2_) used in traditional memristor which makes it suitable for multimodal (optical/electrical) resistive switching, with adding a layer of functionality beyond purely electrical control.^[^ ^1^, ^17^ ^]^ Therefore, their intriguing electronic characteristics, such as the optical and electrical tuning of the conductance state, which makes them suitable for potential applications in nonvolatile memory systems and provide a foundation for high‐performance neuromorphic computing applications and in‐sensor computing systems.

Using Sb_2_S_3_ thin films for multilevel conductance through electrical and optical modulation presents a promising frontier in the field integrated sensory artificial synaptic devices.^[^ ^18^ ^]^ Hence, this study leverages the vapor transport deposition (VTD) method to clarify the intricate conductive properties and memory functionalities of Sb_2_S_3_ thin films, thereby paving the way for next‐generation memory devices with enhanced performance and scalability. The choice of the VTD method is pivotal in the fabrication process with a high deposition rate, enabling precise control over the film thickness and structural properties.^[^ ^19^ ^]^ The inherent advantages of this VTD technique provides a platform for systematically investigating and harnessing the unique attributes of Sb_2_S_3_ thin films to achieve multiple conductance levels within a single device architecture.^[^ ^20^ ^]^ Further, the exploration of multilevel conductance modulation in thin films has profound implications for memory applications.^[^ ^21^ ^]^ Encoding and storing multiple conductivity states within a single‐material system opens avenues for high‐density memory architectures, low‐power‐consumption devices, and robust data storage solutions.^[^ ^22^ ^]^

This study integrates empirical investigations with the theoretical framework of the Sb_2_S_3_ thin‐film behavior to elucidate fundamental mechanisms governing its conductive states. The objective of this study establish a viable pathway for implementing advanced memory technologies. This study presents a novel VTD technique for Sb_2_S_3_ thin film deposition and its elemental and optical characterizations. In addition, exploring Ag/Sb_2_S_3_/Pt crossbar memristor devices for multilevel electrical conductance modulation emphasizes their potential for synaptic applications. The Ag/Sb_2_S_3_/Pt memristor device was used for optical switching. This research delves into an intricate interplay between electrical and optical modulations, providing insights into the transformative effect on next‐generation electronic memory devices. This study contributes to a comprehensive understanding of Sb_2_S_3_ thin films by bridging experimental findings with theoretical insights, paving the way for their effective utilization in shaping the evolving landscape of advanced memory technologies.

## 2 Results and Discussion

### 2.1 Fabrication and Physiochemical Characterization of Ag/Sb_2_S_3_/Pt Memristors

**Figure 1:**
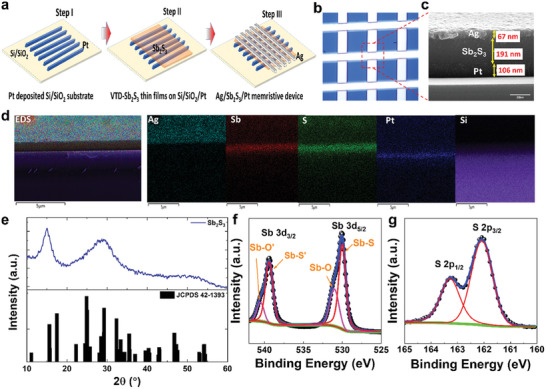
a) Illustration of the fabrication process of Sb_2_S_3_ crossbar array. b) Optical image of a fabricated vertical stack of the array. c) SEM cross‐sectional stack of the Ag/Sb_2_S_3_/Pt memristor device. d) SEM‐EDS elemental mapping of the cross‐sectional stack with Ag as the top electrode, Sb_2_S_3_ as an active layer, and Pt as a bottom electrode. e) XRD pattern of VTD‐deposited Sb_2_S_3_ with a JCPDS card. The XPS analysis of the VTD‐deposited Sb_2_S_3_ with matching binding energies of f) Sb and g) S.

Sb_2_S_3_ thin films were investigated to confirm their chemical and structural configurations before elucidating the electrical characteristics of Sb_2_S_3_. **Figure** **1**a illustrates the crossbar fabrication process for the VTD‐Sb_2_S_3_ thin‐film‐based Ag/Sb_2_S_3_/Pt memristor, and Figure 1b depicts the optical image of a fabricated vertical stack of the array. The thickness of each layer in the crossbar structure was determined using a cross‐sectional SEM image of a stack of Ag/Sb_2_S_3_/Pt memristors or a single‐crossbar device (Figure 1c). This offers a detailed view of the different layers and thickness of each layer. The observed thickness of the top layer (Ag), sandwiched layer (Sb_2_S_3_), and bottom layer (Pt) is ≈67, 191, and 106 nm, respectively (Figure 1c). This information provides a profound understanding of nanoscale dimensions and the overall structure of the fabricated crossbar architecture. These precise measurements underscore the meticulous control exerted during the fabrication process, which influences the functional properties of the device. Nanoscale thickness values are critical parameters that affect the electrical and mechanical behaviors of the crossbar structure and influence its performance in diverse applications.^[^ ^23^ ^]^ Elemental analysis was conducted using SEM‐EDS mapping (Figure 1d). SEM‐EDS mapping offers insights into the elemental distribution within the crossbar structure. The mapping results revealed the homogeneous deposition of Ag over the Sb_2_S_3_ and Pt layers, indicating a uniform distribution of these elements within the analyzed region. This observation underscores the precision of the fabrication process and affirms the successful creation of a Ag/Sb_2_S_3_/Pt memristor with a consistent elemental composition across the nanoscale structure. A uniform distribution of elements is necessary for ensuring reliable and reproducible device performance, as it affects the electrical and material properties required for the functioning of the memristor.^[^ ^24^ ^]^

The XRD pattern of the as‐prepared Sb_2_S_3_ illustrated in Figure 1e reveals its amorphous nature, with broadening peaks corresponding to the crystalline phase of JCPDS card 42–1393. Instead, the pattern is consistent with that of a previously reported amorphous Sb_2_S_3_ material.^[^ ^25^ ^]^ This observation confirmed that the synthesized Sb_2_S_3_ in this study lacked a distinct crystalline structure, as supported by the absence of characteristic peaks in the XRD pattern. This match with previously reported amorphous Sb_2_S_3_ further validates the consistency and reproducibility of the synthesis method. In addition, the elemental composition and valence state of the VTD‐deposited Sb_2_S_3_ were analyzed using XPS. The XPS survey spectrum shows peaks of Sb, S, O, and C at the surface of the VTD‐deposited Sb_2_S_3_ film. The deconvolution of peaks in Figure 1f at 538.9 and 529.7 eV can be indexed to the binding energies of Sb 3d_3/2_ and 3d_5/2_, indicating the presence of Sb^3+^.^[^ ^26^ ^]^ The doublet signal observed in the Sb 3d spectra is attributed to the oxide species (Sb_2_O_3_) at a higher binding energy (pink), and as expected, to Sb_2_S_3_ at a lower binding energy (red). The significant intensity of the sulfide component indicates a higher concentration of Sb_2_S_3,_ whereas the presence of a shoulder oxide pack is attributed to sulfur deficiency during synthesis. The peaks for S 2p at 163.2 and 162 eV (Figure 1g) are assigned to S 2p_1/2_ and S 2p_2/3_, respectively, in the oxidation state of −2.^[^ ^27^ ^]^ The morphology or surface topography of the deposited Sb_2_S_3_ was characterized by atomic force microscopy (AFM). Figure S1a–c (Supporting Information) show 2D AFM images (10 µm × 10 µm) at three different locations of the same Sb_2_S_3_ thin film, with RMS values (surface roughness) of 2.9, 2.2, and 2 nm. These RMS values indicate uniformity in the surface roughness of the Sb_2_S_3_ thin film. For better understanding, Figure S1d–f (Supporting Information) shows 3D AFM images of the Sb_2_S_3_ thin film. Therefore, the sputtering technique is believed to effectively produce a uniform amorphous Sb_2_S_3_ film.

### 2.2 Electrical Optimization and Multilevel Conductance

**Figure 2:**
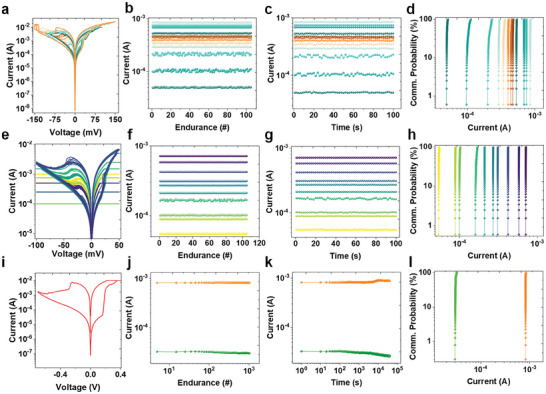
a) Bias‐dependent multilevel I–V characteristics of VTD deposited Sb_2_S_3_ memristors, b) Cyclic endurance of each state, and c) Nonvolatile retention behavior of each state with d) Distinctly different cumulative probabilities of each state. e) Compliance‐current‐dependent growth controlled multilevel I–V characteristics in devices with f) Cyclic endurance of each state, and g) Non‐volatile retention behavior of each state with h) distinctly different communitive probabilities of each state. i) I–V characteristics at optimized measurement conditions for reproducibility at 0.4 to −0.7 V potential window of Ag/Sb_2_S_3_/Pt devices with 50 µm radius. j) Endurance and k) Retention measured at optimized conditions. l) Communitive probabilities of ON and OFF states under optimized conditions.

In the realm of memory‐storage applications our research on memory encompasses a comprehensive examination of the current–voltage (I–V) characteristics inherent in the fabricated crossbar architecture of the Ag/Sb_2_S_3_/Pt memristor device. This investigation is motivated by the prominent challenge in mitigating power consumption, which is a critical consideration within the landscape of memory devices.^[^ ^28^ ^]^ Specifically, we focused on implementing memristors, where the optimization of operational parameters (i.e., the operating voltage) has emerged as a pivotal avenue for enhancing efficiency. **Figure** **2**a presents the voltage‐dependent characteristics in the range of ±0.1–1.5 V, clarifying the multilevel switching phenomenon in crossbar Ag/Sb_2_S_3_/Pt memristors. Remarkably, the observed behavior underscores the capability of the device to undergo consistent and reliable switching across the entire spectrum of measured potentials. Deliberate variation in applied voltages reveals a systematic and controlled modulation of memristive states, which demonstrates the ability of the device to exhibit multilevel switching responses.^[^ ^29^ ^]^ The uniformity of the switching behavior across the entire voltage range provides evidence for the reliability of the device. Remarkably, the operational potential of the device is low (< ±1 V), which contributes to its energy efficiency.^[^ ^18a^ ^]^ Further, endurance, which reflects the cyclic stability of the memory devices, is an important property of the device. Our memristor demonstrates considerable endurance, emphasizing its potential suitability for long‐term and reliable memory applications. The multilevel endurance of the manufactured device, comprising numerous resistance states at different bias voltages, is shown in Figure 2b. In addition, as shown in Figure 2c, we measure the retention for different biases to ensure that the device can maintain the resistance state and data over time. The cumulative probability graph of multiple resistances is illustrated in Figure 2d, demonstrating the distribution of resistance states at a specific bias and delineating one high‐resistance state (HRS) and multiple low‐resistance states (LRS). We measured the I–V characteristics under an operating bias in the range of 0.1–0.4 for comprehending the minimum potential at which the device demonstrates a notably higher ON/OFF ratio and an expanded memristive area (Figure S2a, Supporting Information). The results revealed a substantial ten‐fold increase in both the ON/OFF ratio and memristive area, as clearly illustrated in Figure S2b (Supporting Information). This enhancement underscores the improved performance of the device for switching efficiency and memristive capabilities within the specified operating bias range. Figure S2c (Supporting Information) shows that the SET voltage remained constant, although variations in the RESET value were noted at various negative bias voltages (−0.2, −0.4, and −0.7 V). In vertical devices, the top electrode radius is a parameter that has a significant effect on memristive features when fabricating memory devices. With this in mind, we examined the I−V characteristics of two different devices with radii (50 and 100 µm) (Figure S2d, Supporting Information). A notable decrease in the current was observed when the device radius was extended. The memristive area and ON/OFF ratio were similarly affected by the device radius (Figure S2d, Supporting Information). The ON/OFF ratio and memristive area decreased by approximately 10 times when the radius increased (Figure S2e, Supporting Information). The I‐V characteristics and cyclic stability of these devices were examined at various potential windows, as depicted in Figure S2f,g (Supporting Information). This figure indicates the variation in SET and RESET voltages corresponding to changes in the potential window. While the device demonstrates a consistent cyclic response when measured within the same potential window. A more detailed explanation is provided in supporting information. The device‐to‐device and cycle‐to‐cycle stability are important for understanding the diversity in switching characteristics and the reproducibility of the fabricated devices. We examined the I–V characteristics up to 10 cycles for the devices with radii of 50 and 100 µm (Figures S3 and S4, Supporting Information). The SET‐RESET potential and I–V characteristics of all devices are nearly identical. Devices with a radius of 50 µm display gradual (analog) RESET, whereas those with a radius of 100 µm display abrupt (digital) RESET.

Multilevel RS can be achieved by appropriately adjusting current compliances.^[^ ^30^ ^]^ We subjected the Ag/Sb_2_S_3_/Pt crossbar memristor device to various current compliances (CC) and analyzed the corresponding I–V characteristics for harnessing the full potential of multilevel RS, as shown in Figure 2e. Across different CC levels, a discernible trend emerged in the improvement of RS, accompanied by concurrent resistance modulation. Interestingly, both polarities exhibited CC‐dependent RS modulation, highlighting the versatility of the device. Cyclic endurance over 100 cycles (Figure 2f) and the nonvolatile retention behavior of each state dependent on CC are presented in Figure 2g. The variability in each resistance state was meticulously examined by plotting cumulative probability graphs, revealing a linear progression in resistance states without any observable degradation, as shown in Figure 2h. This underscores the reliability and consistency of the device in maintaining different resistance states over repeated cycles. Furthermore, I–V characteristics measured at the optimized measurement conditions within the 0.4 to −0.7 V potential window of Ag/Sb_2_S_3_/Pt device with 50 µm radius for reproducibility are depicted in Figure 2i. The device showed consistent endurance over 10^3^ cycles under optimized conditions, as shown in Figure 2j, and a long retention time of up to 4 × 10^4^ s, as shown in Figure 2k. Cumulative probability graphs are used to carefully analyze the variability in each resistance state. The results indicated that the resistance states progressed linearly without showing any signs of degradation (Figure 2l).

### 2.3 Device to Device and Cycle to Cycle Stability

**Figure 3:**
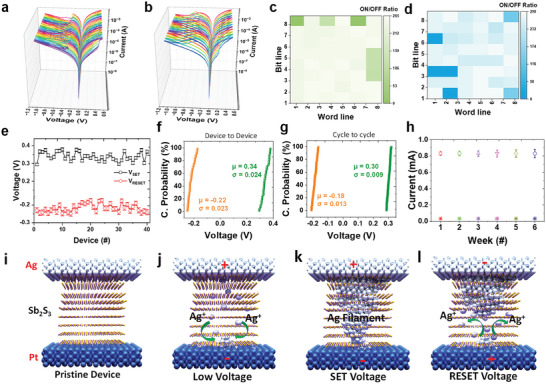
*I–V* switching of all working devices on fabricated a) batch 1 and b) batch 2. c) ON/OFF ratios of all working devices on fabricated d) batch 1 and e) batch 2. (e) Switching voltage variation across all devices. f) Cumulative probability of device‐to‐device switching voltage of batch 1. g) Cumulative probability of the cyclic variability of switching voltages. h) Reliability of devices in the ON and OFF state through the course of the six weeks. Plausible resistive switching (RS) mechanism for Ag/Sb_2_S_3_/Pt memristive device. i) Pristine device, j) device at low voltage, k) SET device, and l) RESET device.

Within this work, we focused on evaluating the reproducibility and performance consistency of a crossbar Ag/Sb_2_S_3_/Pt memristor device. Two distinct batches of the device were manufactured (Batch 1 and Batch 2) to ensure a comprehensive assessment. The I–V characteristics of all operational devices from both batches are analyzed meticulously and presented visually in **Figure** **3**a,b. This examination provides insights into the electrical behavior of memristor devices, thereby forming the foundation for subsequent evaluations. We investigated the device‐to‐device ON/OFF ratio variations for both batches to gauge the reliability of the fabrication process, as shown in Figure 3c,d. Impressively, our findings revealed that nearly 90% of the devices in both batches exhibited ON/OFF ratios exceeding 200. This remarkable consistency suggests robust productivity and reproducibility in the fabrication process, reinforcing the credibility of the device for practical applications.^[^ ^12b^ ^]^ As shown in Figure 3e, we explored the SET and RESET voltage variations for a subset of 40 devices to further investigate the performance characteristics. Furthermore, Figure S10 (Supporting Information) depicts the variation in SET and RESET voltages for all working devices in Batch 1 and Batch 2 within the crossbar structure. The cycle‐to‐cycle and device‐to‐device performances for Batch 1 are shown in Figures S5–S8 (Supporting Information), and for Batch 2 in Figure S9 (Supporting Information). The cumulative probability of the device‐to ‐device switching voltages for Batch 1 is shown in Figure 3f, and the cyclic variability of the switching voltages is illustrated in Figure 3g. These analyses underscore the consistent performance observed across different devices in Batch 1, emphasizing the reliability and uniformity of the switching behavior of the device. Maintaining consistent performance over an extended period is a demanding technological requirement for memory applications. Figure 3h demonstrates the ability of the fabricated device to retain a consistent current state after six weeks. This extended term stability confirms the potential of this device for reliable long‐term memory applications, highlighting its practical viability in real‐world scenarios. This paper presents a thorough investigation of the reproducibility and performance consistency of a crossbar Ag/Sb_2_S_3_/Pt memristor device. The comprehensive analysis of the I–V characteristics, ON/OFF ratios, SET and RESET voltage variations, and long‐term stability collectively affirm the outstanding stability and reproducibility of the fabricated device. These findings suggest that the crossbar Ag/Sb_2_S_3_/Pt memristor is a promising candidate for reliable and enduring memory applications, with potential implications for advancements in the field of electronic memory devices.

Based on the I–V characteristics, we suggest a possible RS mechanism for the Ag/Sb_2_S_3_/Pt memristive devices, as shown in Figure 3. When there is no initial bias, the device is in the HRS, as shown in Figure 3i. The Ag^+^ ions are generated when the Ag metal oxidizes in response to a positive bias applied to the top Ag electrode (Ag → Ag^+^ + e^−^), as shown in Figure 3j. Subsequently, the applied electric field causes the Ag+ ions to migrate in the direction of the negatively biased Pt electrode.^[^ ^31^ ^]^ The conductivity of the material increased significantly when the concentration of Ag^+^ ions reached a threshold value. Then, the Ag^+^ ions were reduced back to Ag at the negatively charged Pt electrode with the formation of an Ag conduction filament, as shown in Figure 3k. Consequently, the device experiences an easy current flow, triggering the SET process, which turns the device from an HRS to an LRS. However, when an opposing bias is applied to the top Ag electrode, the reduced Ag metal species ionized again to Ag^+^ and attempted to migrate towards the negatively biased top electrode, breaking the created conduction route and initiating the RESET process, as shown in Figure 3l.^[^ ^3^, ^32^ ^]^ The detailed possible RS mechanism during the SET and RESET process is depicted and explained in supporting information Figure S11 (Supporting Information).

### 2.4 Electrical Synaptic Properties of Ag/Sb_2_S_3_/Pt Memristor

**Figure 4:**
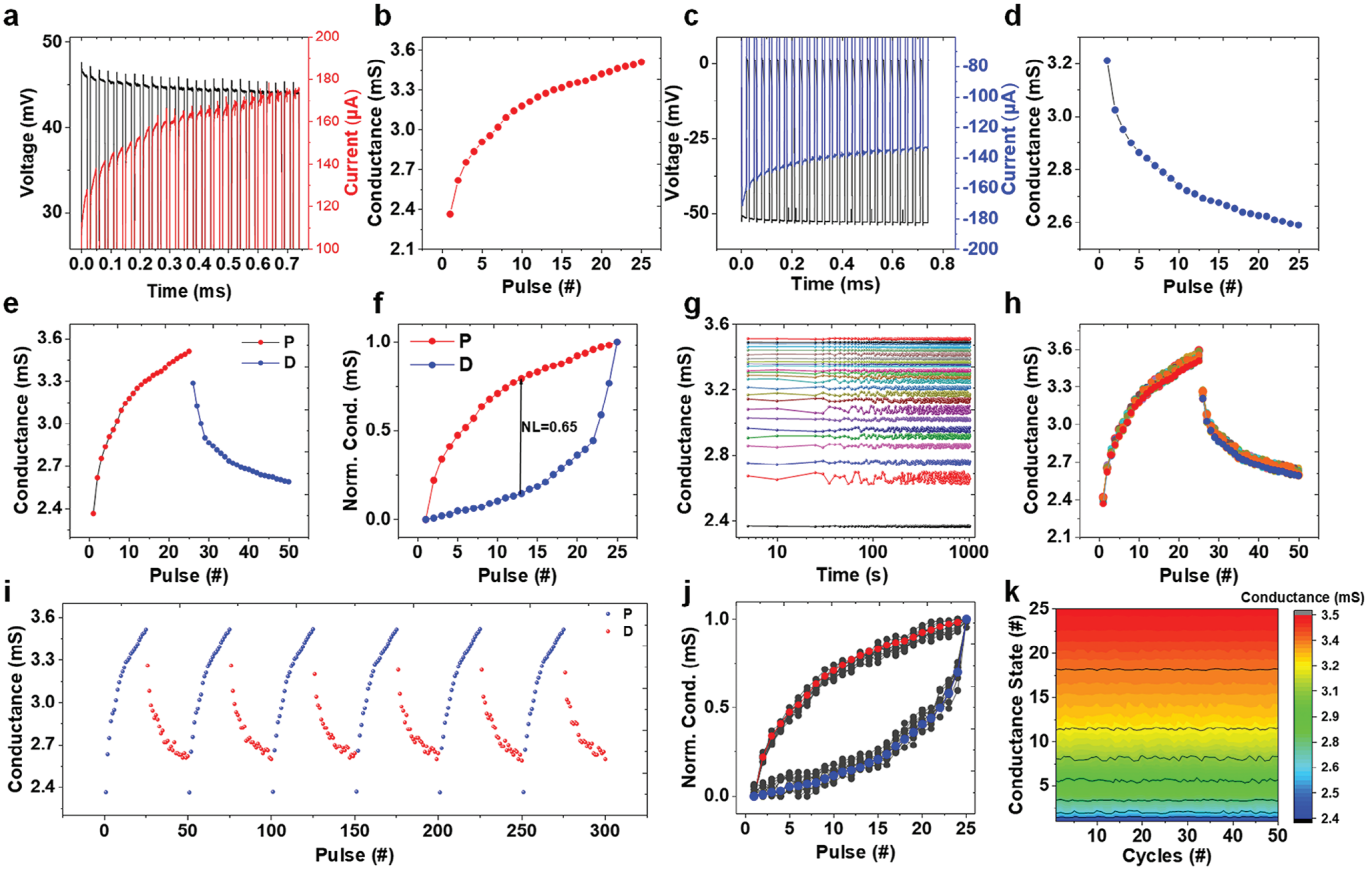
a) Excitatory postsynaptic current (EPSC) electrical pulsed potentiation responses of Sb_2_S_3_ devices and b) learning/potentiation behavior in conductance states. c) Inhibitory postsynaptic currents (IPSC)‐electrical inhibitory responses of Sb_2_S_3_ devices and d) forgetting/depression behavior in conductance states. e) Representation of successive potentiation and depression of the Ag/Sb_2_S_3_/Pt memristor devices. f) Nonlinearity of the P–D behavior. g) Nonvolatile behavior of each conductance states in the potentiation process. h) Device‐to‐device reproducibility of the P–D characteristics measured across ten devices. i) Cyclic reproducibility of learning and forgetting behaviors measured up to 300 successive pulses. j) Device‐to‐device reproducibility of nonlinearity characteristics measured across ten devices. k) Cyclic reproducibility of randomly modulated state through electrical writing.

Neurotransmitters form synapses that link neurons, facilitating the transmission of signals between them and resulting in information processing.^[^ ^33^ ^]^ This process is simulated in artificial synaptic devices using conductance for indicating synaptic weight and electrodes to represent synaptic terminals. Electrical signals can be used for changing this weight to simulate synaptic plasticity, which plays a vital role in memory and learning.^[^ ^34^ ^]^ This dynamic process is pivotal for the brain's learning and memory capabilities.^[^ ^35^ ^]^ We measured the potentiation (P) and depression (D) characteristics of the fabricated Ag/Sb_2_S_3_/Pt memristor device by applying electrical pulses for emulating the synaptic properties, as illustrated in **Figure** **4**. Further, Figure 4a depicts the excitatory postsynaptic current (EPSC) responses during the electrical pulsed potentiation of the Sb_2_S_3_ device, which reveals an augmented current response with the application constant pulse amplitude at 0.45 V with a constant pulse interval of 20 µs. To optimize this pulse parameter, we tested the device at various pulse amplitudes (0.1–1 V) and observed a considerable increase in the current level at 0.45 V when we changed the pulse duration (0.2 to 1 ms) to the same stimulus voltage, as shown in Figures S12–S14 (Supporting Information). This observed behavior mirrors the learning process/potentiation, where conductance states are achieved with an increase in the pulse number, correlating with the augmentation of the synaptic weight in biological synapses (Figure 4b). Conversely, the fabricated device exhibited a depression behavior indicative of forgetting when subjected to −0.5 V voltage pulses with a 20‐µs pulse interval. The current response for the applied electrical pulse is detailed in Figure 4c, which demonstrates a reduction in the current magnitude with applied negative potential pulses, which is analogous to the reduction in synaptic weights during forgetting in biological synapses.^[^ ^36^ ^]^ Conductance states associated with the depression behavior are presented in Figure 4d. In addition, we performed tests by subjecting the device to successive combined potentiation and depression pulses, measuring their performance, as depicted in Figure 4e. This experiment demonstrates the ability of the device to modulate its conductance by applying consecutive positive and negative potential pulses. Figure 4f illustrates the significant nonlinearity of both potentiation and depression characteristics; the non‐linearity coefficient was 0.65. This substantial non‐linearity poses a potential challenge because it can lead to inaccuracies in pattern recognition after learning, particularly in applications involving image and speech processing.^[^ ^37^ ^]^ The stability of the individual conductance states (weights) is of significant importance in the potentiation and depression processes. Therefore, the nonvolatile behavior of each conductance state during the potentiation process is assessed, as illustrated in Figure 4g. These findings indicate that the conductance states of the Ag/Sb_2_S_3_/Pt memristor device remained stable for up to 10^3^ s, highlighting their potential suitability for commercialization. Furthermore, the device‐to‐device reproducibility of the P–D characteristics was measured across ten devices, as shown in Figure 4h. The corresponding device‐to‐device reproducibility of the nonlinearity characteristics was measured across ten devices (Figure 4j). Almost all ten devices showed similar P–D behaviors and nonlinearity. Then the device was tested for the cyclic reproducibility of the learning and forgetting behaviors for 300 successive pulses (Figure 4i). The cyclic reproducibility of each state modulated randomly by electrical writing is shown in Figure 4k. Consequently, our Ag/Sb_2_S_3_/Pt memristor device emulates synaptic functions with prolonged stability, offering promising prospects for artificial intelligence.

**Figure 5:**
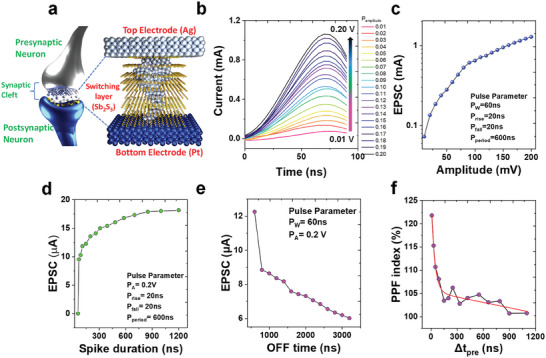
a) Illustration of biological neurons and structural similarity offered by the memristor device structure. b) Writing stimulus amplitude dependence post synaptic responses. c) Stimulus‐amplitude‐dependent EPSC behavior. d) Spike‐duration‐dependent EPSC behavior. e) Spike‐OFF‐time‐dependent EPSC behavior. f) PPF index of exponential decay within the course of the pre‐time.

**Figure** **5**a highlights the structural similarity between the device and biological neurons, suggesting its suitability for neuromorphic applications. In biological neural networks, the presynaptic release of excitatory or inhibitory neurotransmitters that bind to receptors on the post‐synapse elicits either EPSC or inhibitory postsynaptic currents (IPSC).^[^ ^38^ ^]^ Figure 5 provides a comprehensive overview of the synaptic‐like behavior of a memristor device based on EPSC with pulse amplitude, pulse duration, and off time. In particular, initially, the current response was measured based on the stimulus amplitude over time as shown in Figure 5b. The postsynaptic current was modulated with a pulse amplitude from 0.01–0.2 V. The corresponding EPSC as a function of the voltage amplitude is shown in Figure 5c (the pulse parameters are shown in the inset). Similarly, the spike duration‐dependent EPSC behavior was extracted by tuning the spike duration at a constant 0.2 V stimulus voltage, as shown in Figure 5d. The EPSC decreased with an increase in the spike‐off time (Figure 5e). Paired‐pulse facilitation (PPF) represents a characteristic phenomenon in short‐term synaptic plasticity. The PPF enhances the responses elicited by two successive presynaptic spikes, particularly when the second spike closely follows the first. Figure 5f shows the extracted PPF behavior by consecutively applying 0.2 V for 600 ns. The occurrence of PPF is attributed to the time interval between the two stimuli. The neurotransmitters released by the prior stimulus persist in the synaptic cleft when the second stimulus reaches the presynaptic neuron. Consequently, the manifestation of PPF is intricately tied to the temporal interval between the applied stimuli. A maximum PPF index value of 122% is achieved at Δt_pre_ = 10 ns. The PPF index value decreased eventually as Δt_pre_ rises.^[^ ^39^ ^]^ An exponential decay pattern is observed during the pre‐time, thereby emphasizing the intricate synaptic characteristics of the memristor device.

### 2.5 Optoelectrical Synaptic Properties of Ag/Sb_2_S_3_/Pt Memristor

**Figure 6:**
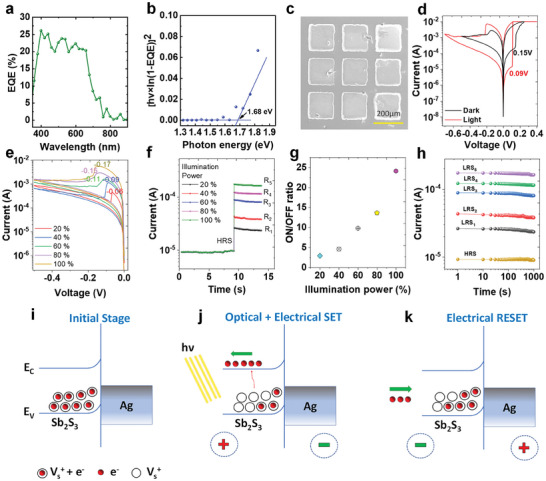
a) Quantum efficiency of the VTD deposited Sb_2_S_3_ thin film and b) bandgap calculation. c) SEM image of device structure utilized for studying optical switching. d) RS behavior in the presence of dark and presence of white light. e) Variation in the RESET voltage with an illumination power f) Nonvolatile nature of each optical state driven by optical switching. g) The ON/OFF ratio based on optical switching with different powers. h) Retention properties of all five LRSs obtained through optical switching. The proposed mechanism for optical switching i) initial state. j) Optical + electrical setting and k) electrical resetting of the devices.

The inherent photosensitive properties of Sb_2_S_3_ make it an intriguing candidate for applications that involve the optical modulation of conductance states. We harnessed this characteristic by implementing an Ag/Sb_2_S_3_/Pt memristor device for optical switching applications. This innovative approach capitalizes on the ability of the material to undergo changes in conductance in response to optical stimuli, opening new avenues for advanced optoelectronic applications. To gain a deeper understanding of the material's response to light, we conducted a comprehensive analysis of the external quantum efficiency (EQE). **Figure** **6**a illustrates the EQE, which amalgamates both optical and electrical influences on the performance of the device. The remarkable light absorption capacity of the fabricated Sb_2_S_3_ films was evident, spanning the UV to the visible spectrum. This broad absorption capability makes the device a versatile tool for optical modulation over a wide range of wavelengths. We further characterized the Sb_2_S_3_ material by utilizing the EQE data to extract the band gap, which revealed a value of 1.68 eV (Figure 6b). This information is pivotal for understanding the active electromagnetic radiation region of a material and providing insights into its optical properties.

Based on this knowledge, we deliberately selected an optical source with a wavelength range of 400–700 nm (white light) for the subsequent experiments, aligned with the absorption characteristics of the material. For the optical measurements, we used single Ag/Sb_2_S_3_/Pt device rather than crossbar structure to exposure of light on Sb_2_S_3_, the detailed fabrication process mentioned in the experimental section. Figure 6c shows an SEM image of the device structure used to study optical switching. The I–V characteristics were measured under two conditions to quantify the effect of the optical stimulation on the electrical behavior of the device: with optical illumination (light) and without (dark). This comparative analysis allowed us to discern the effect of optical stimuli on the electrical response of the memristor device. The results of these experiments offer significant insights into the dynamic interplay between optical excitation and electrical behavior, paving the way for the development of optically controlled electronic devices.

Figure 6d presents the I–V characteristics of the Ag/Sb_2_S_3_/Pt memristor device under both light and dark conditions, revealing substantial differences in the SET and RESET potentials, ON/OFF ratios, and hysteresis areas. This stark contrast underscores the significant effect of optical switching on the electrical behavior of the device. Specifically, the SET potential experiences a notable decrease from 0.15 to 0.09 V, whereas the RESET voltage undergoes an increase, thereby suggesting a clear effect of optical stimuli on the switching dynamics. We varied the illumination intensity on the RESET voltage to further understand the nuanced relationship between the optical illumination and electrical characteristics. Figure 6e illustrates a consistent escalation in both the RESET voltage and the area under the curve with increasing illumination power. The cause of variation in RESET voltage in Figure 6e is the illumination power because the operating window is the same (0 to −0.5 V). In contrast, the variation in RESET voltage observed in Figure S2f (Supporting Information) was due to changes in the operating potential windows. This systematic investigation offers insights into the correlation between the optical power and the resultant changes in the conductance states of the memristor. Figure 6f presents a detailed investigation across illumination ranging from 20–100%, revealing five distinct resistance states. This observation underscores the substantial modulation of conductance states induced by optical stimuli, thereby offering a deeper understanding of the responsiveness of the device to vary the levels of optical illumination. An assessment of the ON/OFF ratio at different illumination powers is presented in Figure 6g. The consistent increase in the ON/OFF ratio with increasing illumination power highlights the capability of the device to achieve precise and controllable conductance‐state modulation through optical means. This finding has significant implications for applications that require dynamic and responsive electronic behaviors. In order to evaluate the retention properties of the device, we measured the stability of the fabricated device at the HRS and at five distinct LRSs over a 1000s period following modulation by optical illumination (Figure 6h).

The potential mechanisms underlying the optical switching observed in individual Sb_2_S_3_‐based devices are depicted in Figure 6. In the pristine device, trapping sites are present in the Sb_2_S_3_ thin films, as illustrated in Figure 6i. Electrons confined within trapping sites can be excited and mobilized by the intrinsic electric field upon exposure to optical illumination, thereby leading to the generation of positively charged sulfur vacancies (V_s_^+^) and inducing the transition of the device from an HRS to an LRS, as shown in Figure 6j.^[^ ^40^ ^]^ Conversely, electrons were reintroduced into the interfacial trapping sites when the Ag/Sb_2_S_3_/Pt device was subjected to an external negative electrical field, consequently restoring the initial state and causing the device to RESET, as shown in Figure 6k.^[^ ^41^ ^]^

Overall, the fabricated Ag/Sb_2_S_3_/Pt device shows great potential as a candidate for future optoelectronic devices in memory and neuromorphic computing applications. To additionally increase memory density, fabricating nanoscale crossbar structures, where each cell is at the sub‐nanometer scale, could be a viable solution.^[^ ^42^ ^]^ The wafer or atomic scale integration of Sb_2_S_3_ materials can significantly enhance memory density.^[^ ^43^ ^]^ Large‐scale fabrication of these devices can be achieved through simple vapor transport deposition (VTD) and sputtering techniques for the switching layer deposition. And the dens nanoscale top and bottom electrodes are deposited with nanoscale patterns using lithography techniques followed by sputtering. These methods allow for scalable production while maintaining the necessary precision and quality of the switching device. To further improve the speed of the Ag/Sb_2_S_3_/Pt device, several strategies can be employed, such as incorporating materials with higher carrier mobility can reduce the response time, like MXene, quantum dots etc. The interface engineering at the Sb_2_S_3_ and electrode contact can reduce contact resistance which can further improve switching speed.

## 3 Conclusion

The investigation of Sb_2_S_3_ thin films revealed their potential for revolutionizing electronic memory devices, facilitated by the VTD technique, thereby offering precise control over the thickness. These Ag/Sb_2_S_3_/Pt memristors exhibited multi‐level conductance modulation within a single‐device architecture, exhibiting low operational voltages, robust endurance, and reliable switching behavior, which are essential for memory applications. RS is enhanced by adjusting the current compliance, which improves the overall device performance. The reproducibility and stability across different device batches validated their reliability for practical implementation. The Sb_2_S_3_‐based memristors emulate synaptic functions with prolonged stability, and they are promising advancements in neuromorphic computing. Optical switching applications demonstrated significant conductance‐state modulation under optical stimulation by leveraging the photosensitivity of Sb_2_S_3_. This research underscores the potential of Sb_2_S_3_ for next‐generation memory devices and optoelectronic applications, thereby shaping the future landscape of electronics and artificial intelligence given its versatility and performance.

## 4 Experimental Section

### Substrate Preparation

A meticulous dual‐step cleaning process was applied to the Si/SiO_2_ (1.5 × 1.5 cm^2^) substrate prior to depositing Sb_2_S_3_ using the VTD technique. This process included ultrasonication in acetone, followed by a 10‐min ultrasonication in an isopropyl alcohol (IPA) solution. After N_2_ gas drying, a layer of platinum (Pt) was deposited onto the Si/SiO_2_ substrate using a magnetron sputtering technique without a shadow mask by an 8 bars shadow mask. The resulting Si/SiO_2_/Pt substrate served as the foundation for the subsequent deposition of VTD‐Sb_2_S_3_ thin films. This preparatory procedure ensures a pristine substrate surface, which promotes the uniform and controlled growth of Sb_2_S_3_ thin films during the subsequent VTD process.

### Vapor Transport Deposition of the Sb_2_S_3_ Switching Layer

A single‐zone furnace (SRDVF‐LV‐3B‐1608, Korea) was used to deposit the Sb_2_S_3_ switching layer onto a Si/SiO_2_/Pt substrate. The quartz tube, assembled in the furnace, held a ceramic crucible containing 0.1 g of the as‐received Sb_2_S_3_ (iTASCO, 3–12 µm granules, 99.999%) in the center zone. The chamber pressure was maintained at 1 Torr, and Ar gas served as the carrier gas. The Si/SiO_2_/Pt substrate was positioned downstream of the furnace in an area with a temperature lower than that of the evaporation zone, with a source‐to‐substrate distance of 17 cm. The furnace temperature increased gradually to 540 °C, with a deposition rate of 18 °C per minute. The substrate was subjected to Sb_2_S_3_ deposition at 540 °C for 2 min, which resulted in a thin film with a thickness of ≈200 nm. Following the deposition, the chamber was allowed to cool naturally.

### Fabrication of Switching Device

The Ag electrode was deposited and completed the fabrication of the 8 × 8 crossbar array switching device by employing the shadow masking technique (8 bar). A 99.9%‐pure Ag metal target was obtained from LTS Research Laboratories (USA). A 70 nm Ag top‐electrode was deposited meticulously through a metal shadow mask using a magnetron sputtering system under Ar environment conditions (flow rate = 20 sccm, base pressure = ≈2.6 × 10^−6^ Torr, and working pressure = ≈5 mTorr). This step ensures the precise placement of the Ag electrode, which was a critical component. The use of high‐purity Ag contributes to the reliability and stability of the device, promoting consistent performance in subsequent electrical characterizations and assessments. For the optical measurement, a device was fabricated with the same process and parameters by employing only the shadow masking technique to deposit the Ag electrode (200 × 200 µm) with a 50 µm distance on a Si/SiO_2_/Pt substrate.

## Conflict of Interest

The authors declare no conflict of interest.

## Author Contributions

S.S.K. and P.S.P. contributed equally to this work. S.S.K., P.S.P., D.D.K., I.S., P.R.P. prepared the materials for most experimental measurements and analyzed the results. S.S.K., D.D.K., J.H., and J.H.P. conceived and designed the study. S.S.K., I.K.G.D., W.A.L., S.S., J.H. assisted with physiochemical characterization, optical instrumentation. T.D.D., S.Y.N. provided valuable insights for this study. S.S.K., D.D.K., J.H., and J.H.P. prepared the manuscript. All the authors discussed the results and commented on the manuscript. All the authors have revised and commented on this manuscript. J.H. and J.H.P. supervised the study.

## Supporting information

Supporting Information

## Data Availability

The data that support the findings of this study are available from the corresponding author upon reasonable request.
