# Supplementary material for: Multilevel Conductance States of Vapor‐Transport‐Deposited Sb2S3 Memristors Achieved via Electrical and Optical Modulation
Source: Adv Sci (Weinh). 2024 Jul 3;11(32):2405251. doi: 10.1002/advs.202405251 (PMC11348134; doi:10.1002/advs.202405251)
Supplement: Supplementary file 1 — Supporting Information [file ADVS-11-2405251-s001.pdf]

ADVANCED SCIENCE
Open Access

Supporting Information

for *Adv. Sci.*, DOI 10.1002/advs.202405251

Multilevel Conductance States of Vapor-Transport-Deposited Sb_2S_3 Memristors Achieved via Electrical and Optical Modulation

Somnath S. Kundale, Pravin S. Pawar, Dhananjay D. Kumbhar, I. Ketut Gary Devara, Indu Sharma, Parag R. Patil, Windy Ayu Lestari, Soobin Shim, Jihye Park, Tukaram D. Dongale, Sang Yong Nam, Jaeyeong Heo and Jun Hong Park**

Supporting Information

Multilevel Conductance States of Vapor-Transport-Deposited Sb_2S_3 Memristors achieved via Electrical and Optical Modulation

Somnath S. Kundale[1,2 †], Pravin S. Pawar[3, †], Dhananjay D. Kumbhar[4], I Ketut Gary Devara[1], Indu Sharma[3], Parag R. Patil[3], Windy Ayu Lestari[1], Soobin Shim[1], Jihye Park[1], Tukaram D. Dongale[4], Sang Yong Nam[1,2], Jaeyeong Heo[3], Jun Hong Park[1*]*

Somnath S. Kundale, I Ketut Gary Devara, Windy Ayu Lestari, Soobin Shi, Jihye Park, Sang Yong Nam, Jun Hong Park

[1] Department of Materials Engineering and Convergence Technology, Gyeongsang National University, Jinju, Gyeongsangnam-do 52828, Republic of Korea

Somnath S. Kundale, Sang Yong Nam

[2] Research Institute for Green Energy Convergence Technology, Gyeongsang National University, Jinju 52828, Republic of Korea

Pravin S. Pawar, Indu Sharma, Parag R. Patil, Jaeyeong Heo

[3] Department of Materials Science and Engineering, and Optoelectronics Convergence Research Center, Chonnam National University, Gwangju 61186, Republic of Korea

Dhananjay D. Kumbhar, Tukaram D. Dongale

[4] Computational Electronics and Nanoscience Research Laboratory, School of Nanoscience and Biotechnology, Shivaji University, Kolhapur 416004, India

[*]Corresponding author

Email: jheo@jnu.ac.kr

Email: yakte@gnu.ac.kr

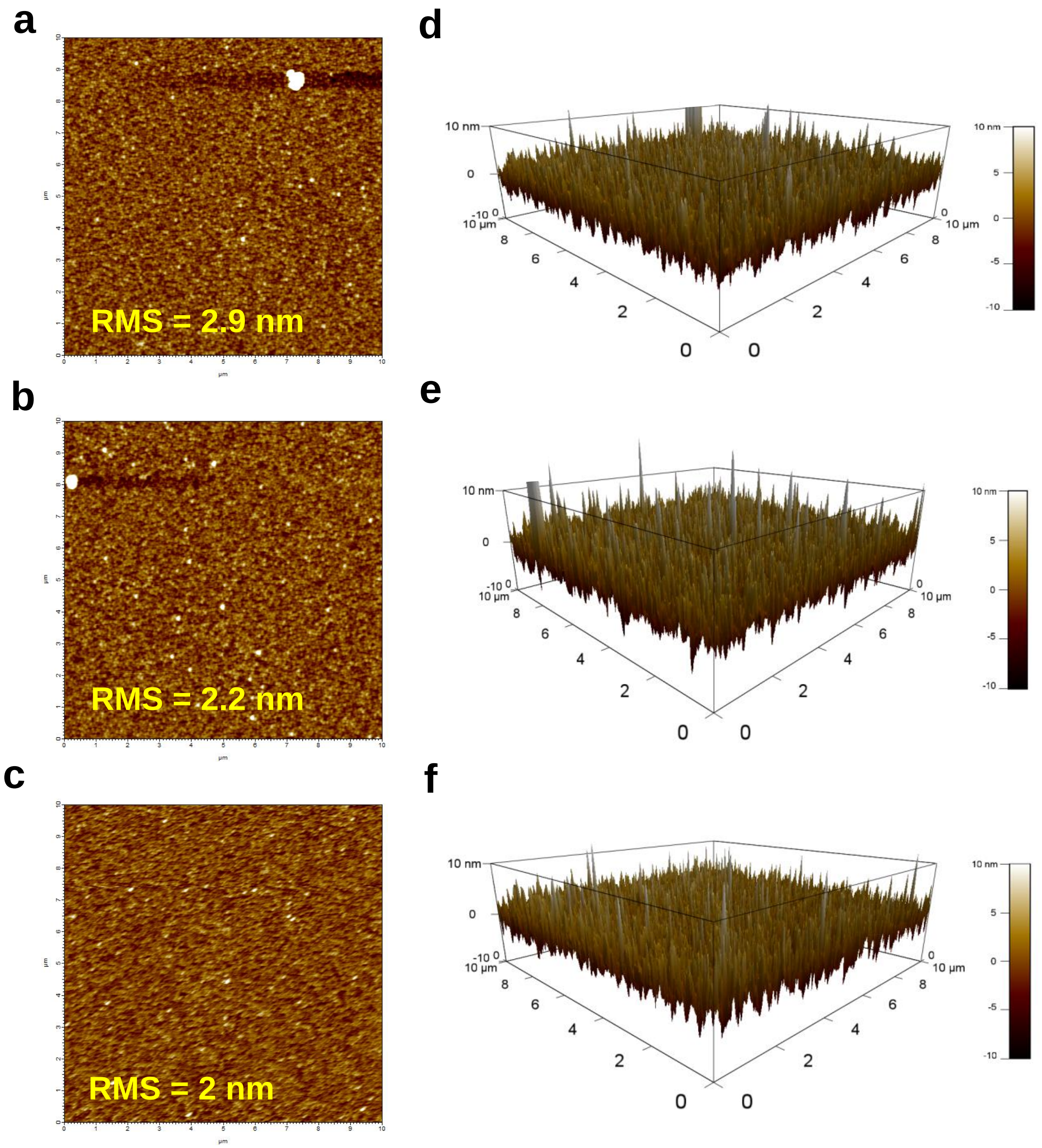

Figure S1 a-c) 2D AFM images (10 μm × 10 μm) at three different locations of the same Sb_2S_3 thin film with their RMS values. **d-f)** 3D AFM images of the Sb_2S_3 thin film at three different locations.

SET, RESET variation and cyclic stability

In analysis of the SET and RESET voltages in Ag/Sb_2S_3/Pt devices with varying radius of top electrodes, we conducted multiple experiments to understand their behavior. As shown in **Figure 2Sc**, distinct SET and RESET voltages were observed at different potential windows. Notably, variations in RESET voltage were observed in devices with 50 µm and 100 µm top electrodes (**Figure 2Sd**). Due to the higher ON/OFF ratio observed in the 50 µm device compared to the 100 µm device, we selected the 50 µm device for further analysis, as indicated in **Figure 2Se**. **Figure 2Sf** presents the I-V characteristics measured within the potential windows of 0.4 to -0.7 V and 0.3 to -0.7 V, respectively. The data demonstrate that the device exhibits a significant memristive response within the 0.4 to -0.7 V potential window, which we identified as the optimized window. Additionally, **Figure 2Sg** depicts the cyclic stability of the device across different potential windows. The cycle-to-cycle and device-to-device variations in SET and RESET voltages are illustrated in **Figures S3** and **S4.** In summary, the minimal cycle-to-cycle variation observed in this device underscores its potential suitability for memory applications.

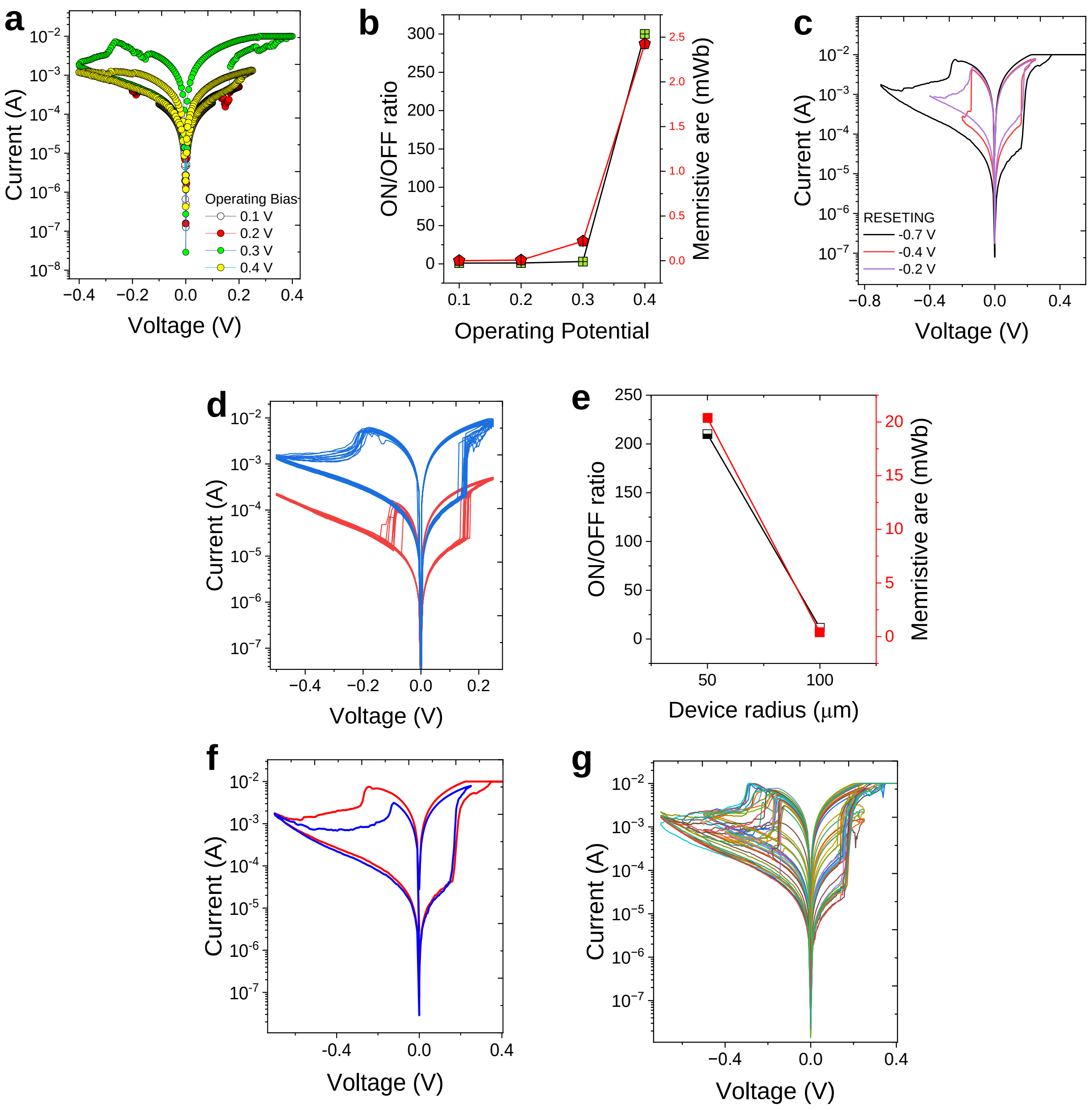

Figure S2 a) Ag/Sb_2S_3/Pt memristor device I-V characteristic measurements at an operating bias ranging from 0.1 to 0.4. **b)** ON/OFF ratio and memristive area of Ag/Sb_2S_3/Pt memristor device at an operating bias ranging from 0.1 to 0.4. **c)** Variation in SET and RESET voltage as increase with switching potentiation. **d)** I-V characteristics of two different device radii (50 and 100µm) **e)** ON/OFF ratio and memristive area of device radii (50 and 100µm) devices. **f)** I-V characteristics (red and blue) measured within the potential windows of 0.4 to -0.7 V and 0.3 to -0.7 V, respectively of 50 µm device. **g)** The device's cyclic stability at different potential windows indicates variations in SET and RESET voltage with operating potential window.

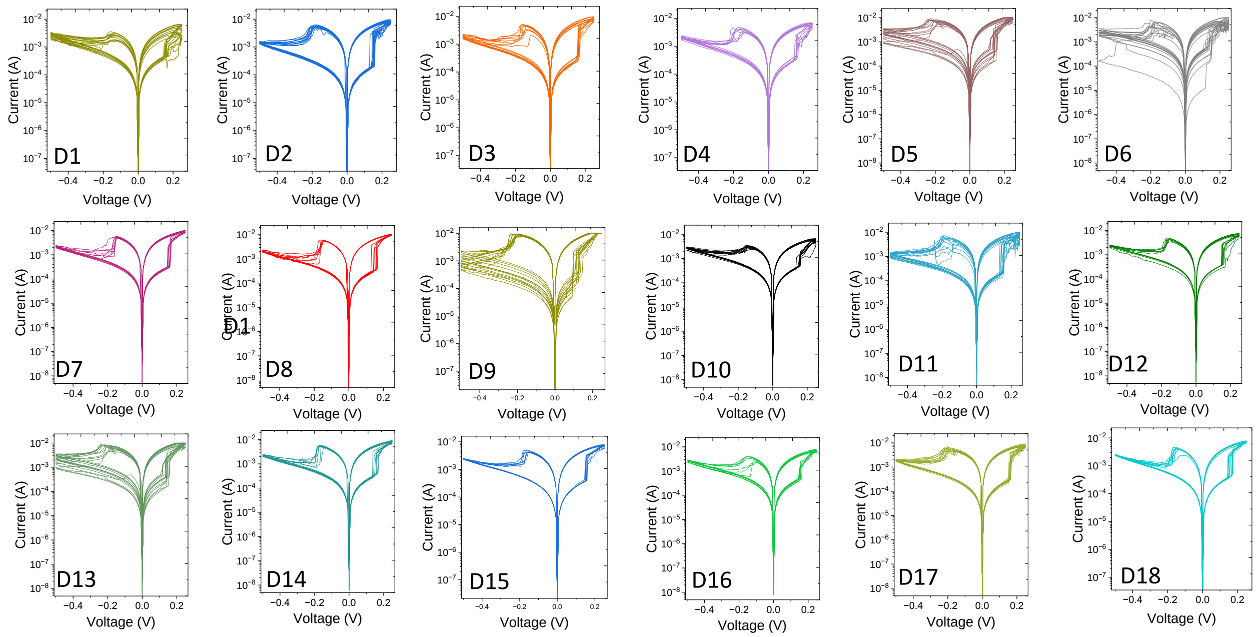

Figure S3 Device to device and cycle to cycle (10 cycles each) stability of $Ag/Sb_2S_3/Pt$ memristor device with 100μm device radius.

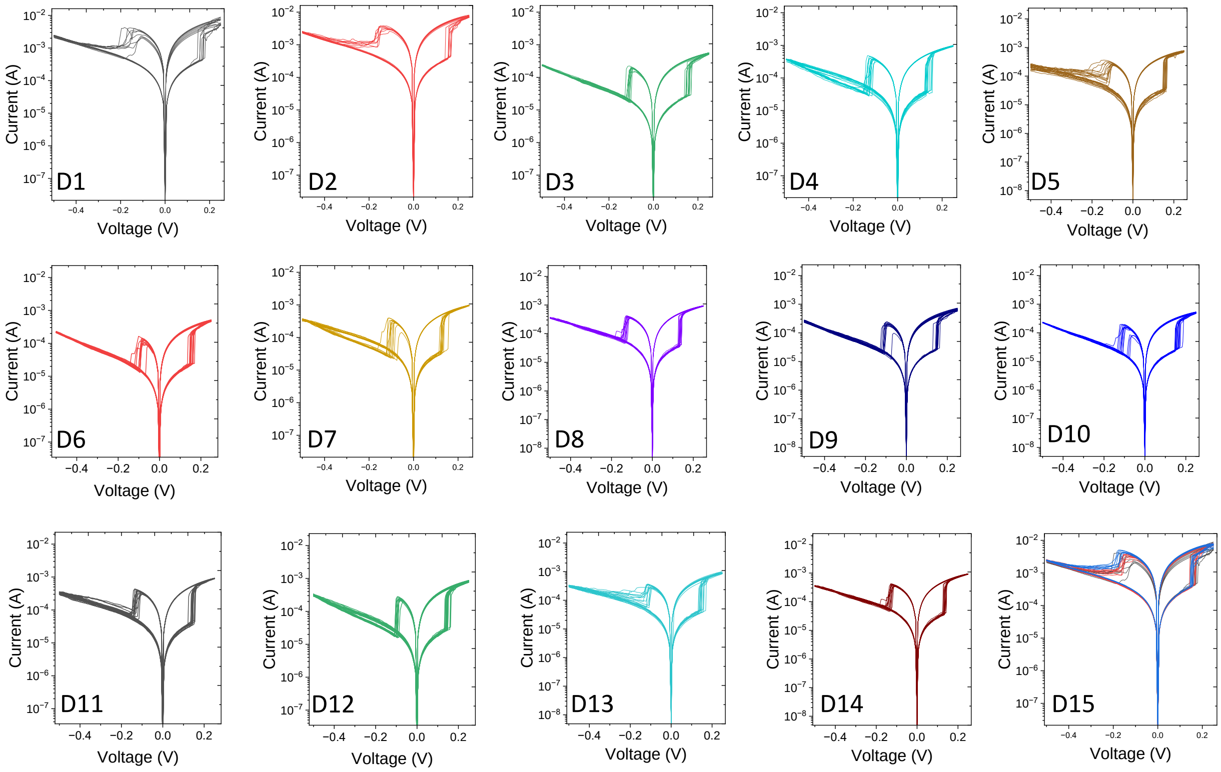

Figure S4 Device to device and cycle to cycle (10 cycles each) stability of $Ag/Sb_2S_3/Pt$ memristor device with 100μm device radius.

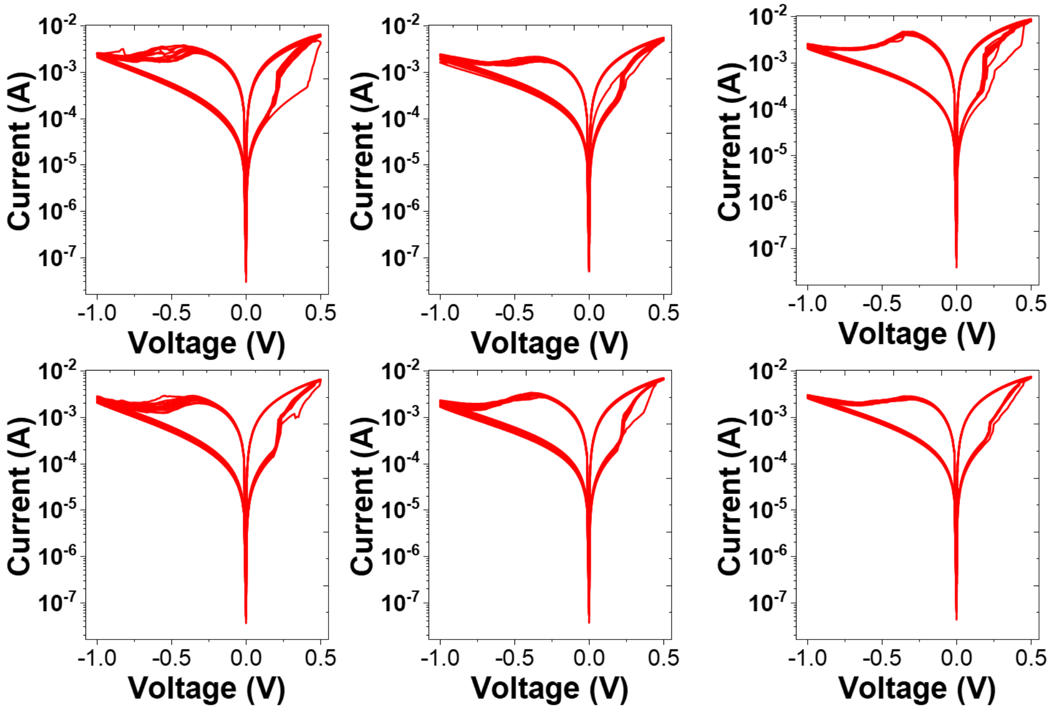

Figure S5. Batch 1, cycle to cycle and device to device performance of $Ag/Sb_2S_3/Pt$ crossbar devices (1-6 devices).

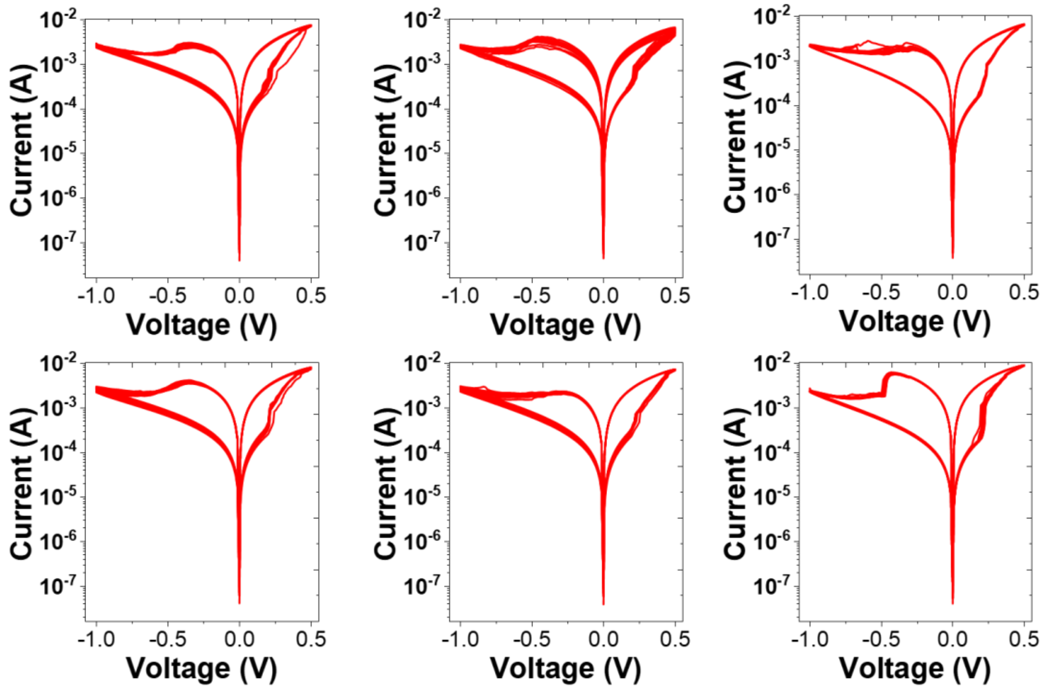

Figure S6. Batch 1, cycle to cycle and device to device performance of $Ag/Sb_2S_3/Pt$ crossbar devices (7-12 devices).

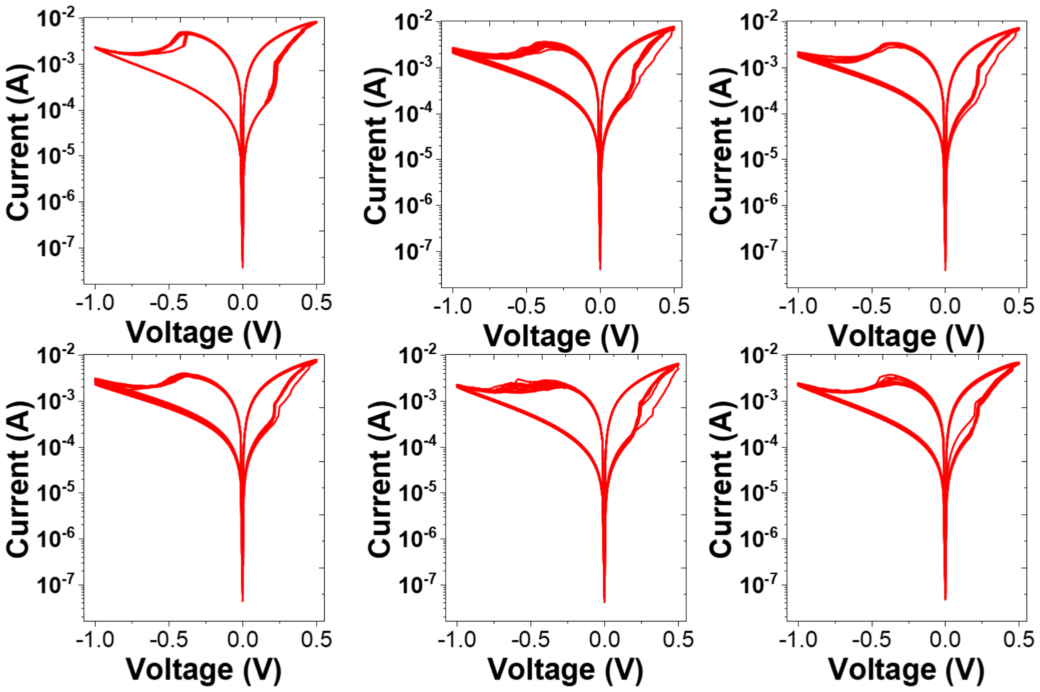

Figure S7. Batch 1, Cycle to cycle and device to device performance of $Ag/Sb_2S_3/Pt$ crossbar devices (13-18 devices).

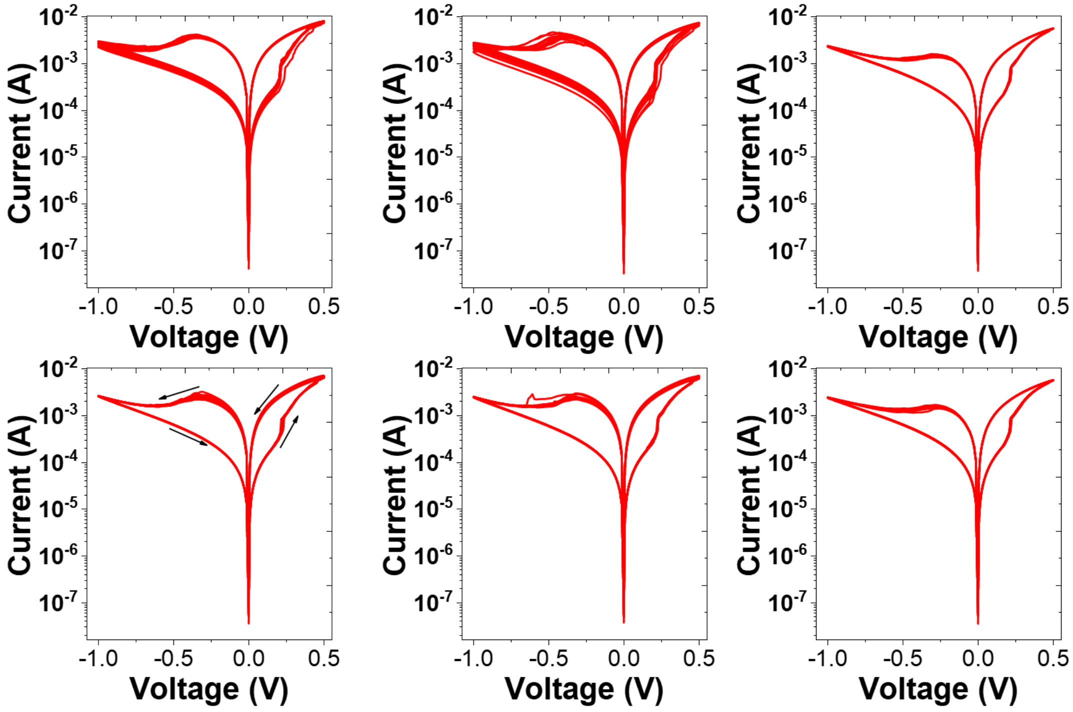

Figure S8. Batch 1, Cycle to cycle and device to device performance of $Ag/Sb_2S_3/Pt$ crossbar devices (19-24 devices).

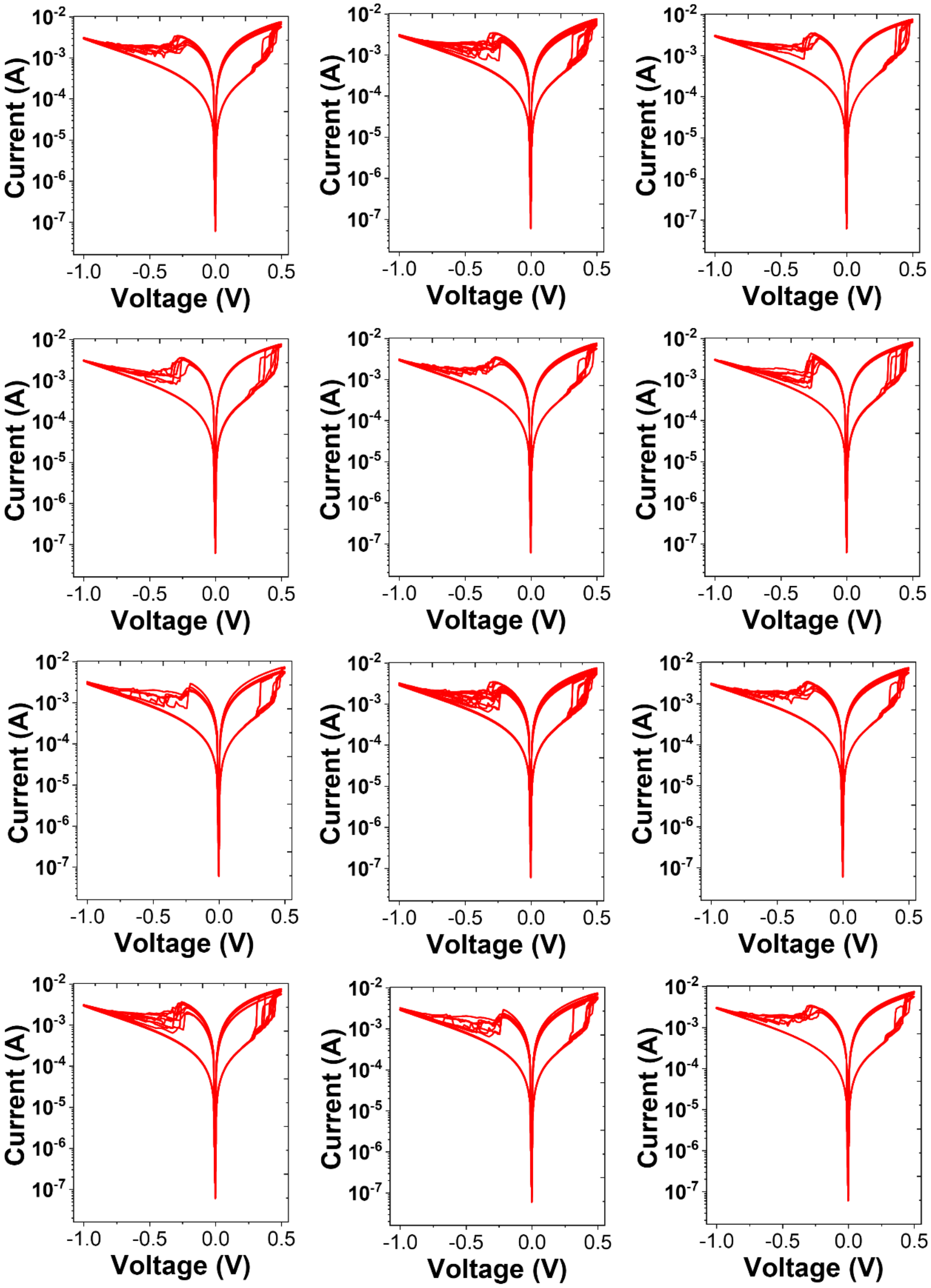

Figure S9. Batch 2, Cycle to cycle and device to device performance of $Ag/Sb_2S_3/Pt$ crossbar devices (1-12 devices).

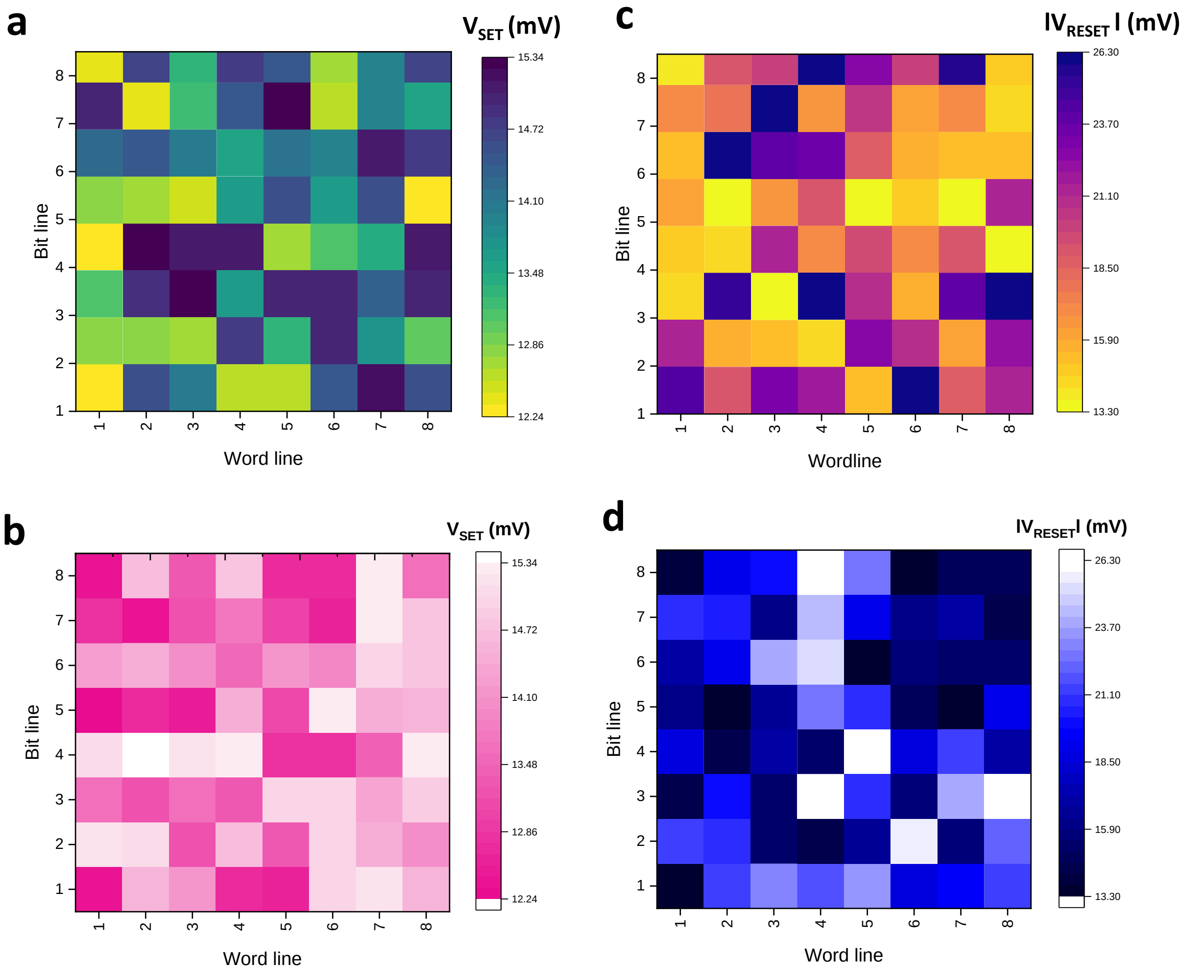

Figure S10. (a-b) SET voltage variation of all working devices on fabricated batch 1 and batch 2. **(c-d)** RESET voltage variation of all working devices on fabricated batch 1 and batch 2.

Detailed resistive switching mechanism

In Ag/Sb_2S_3/Pt memristive device, ionizable Ag top electrode plays an important role in resistive switching mechanism. The electrical ionization of the Ag atoms from top electrodes initiates the switching mechanism. At small voltage (0.1V) a very small amount of Ag gets ionized ($Ag \rightarrow Ag^+ + e^-$). As increasing applied positive electrical bias (0.2 and 0.3) higher amount of the Ag get ionized and due to their positive charge, they are migrated toward the grounded Pt electrode and get reduced ($Ag^+ + e^- \rightarrow Ag$) to form the conduction path (Ag filament). At the SET voltage the complete conduction filament is formed which switches the device to low resistance state (LRS). The applied bias dependent growth of the conduction path is depicted in the SETTING process of **Figure S11**. In contrast, the application of increasing negative bias (from -0.2 V, -0.3 V, etc.) gradually dissolves the Ag filaments due to ionization of conduction filaments Ag atom ($Ag \rightarrow Ag^+ + e^-$), initiating the RESET process. The complete rupture of Ag filaments switches the device back to the high resistance state (HRS). The detailed process of RESETTING is depicted in **Figure S11**.

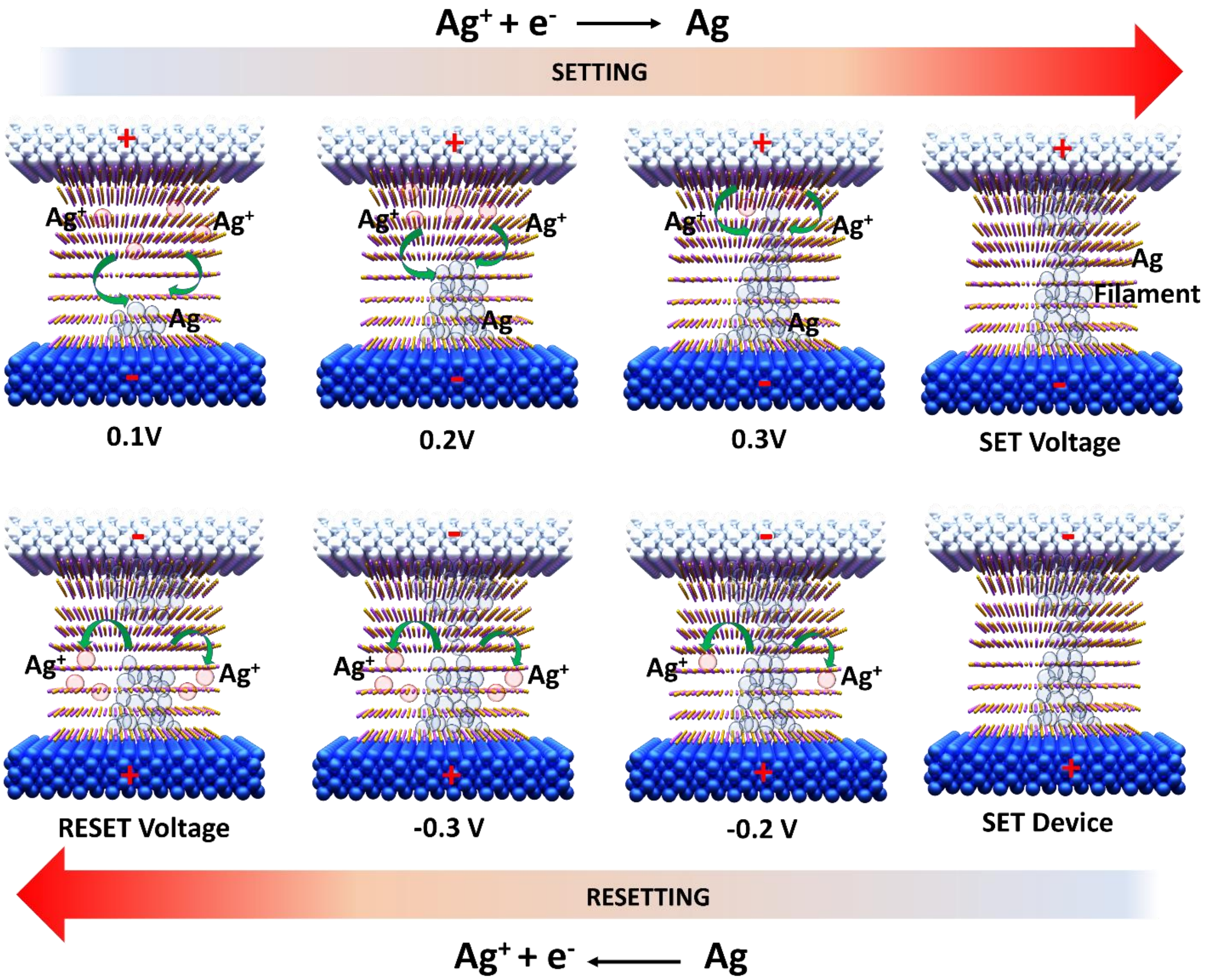

Figure S11. Detailed possible resistive switching mechanism in fabricated Ag/Sb_2S_3/Pt device in both Setting and Resetting cases.

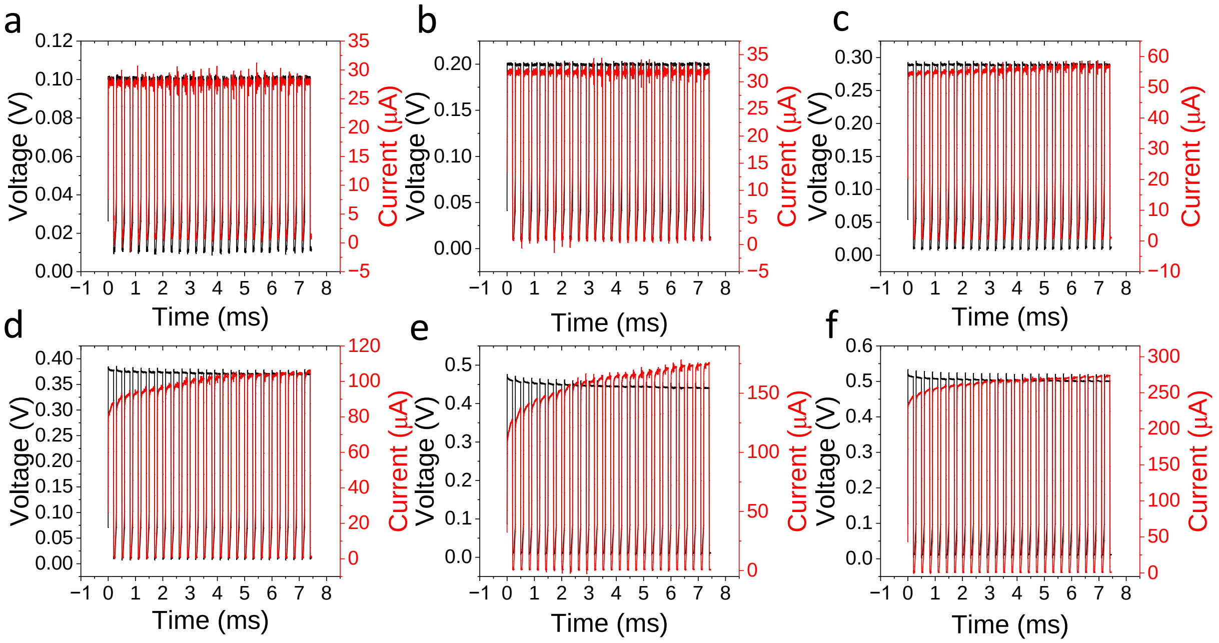

Figure S12. Pulse amplitude modulated synaptic plasticity **(a)** 0.1V **(b)** 0.2V **(c)** 0.3V **(d)** 0.35V **(e)** 0.45V **(f)** 0.5V

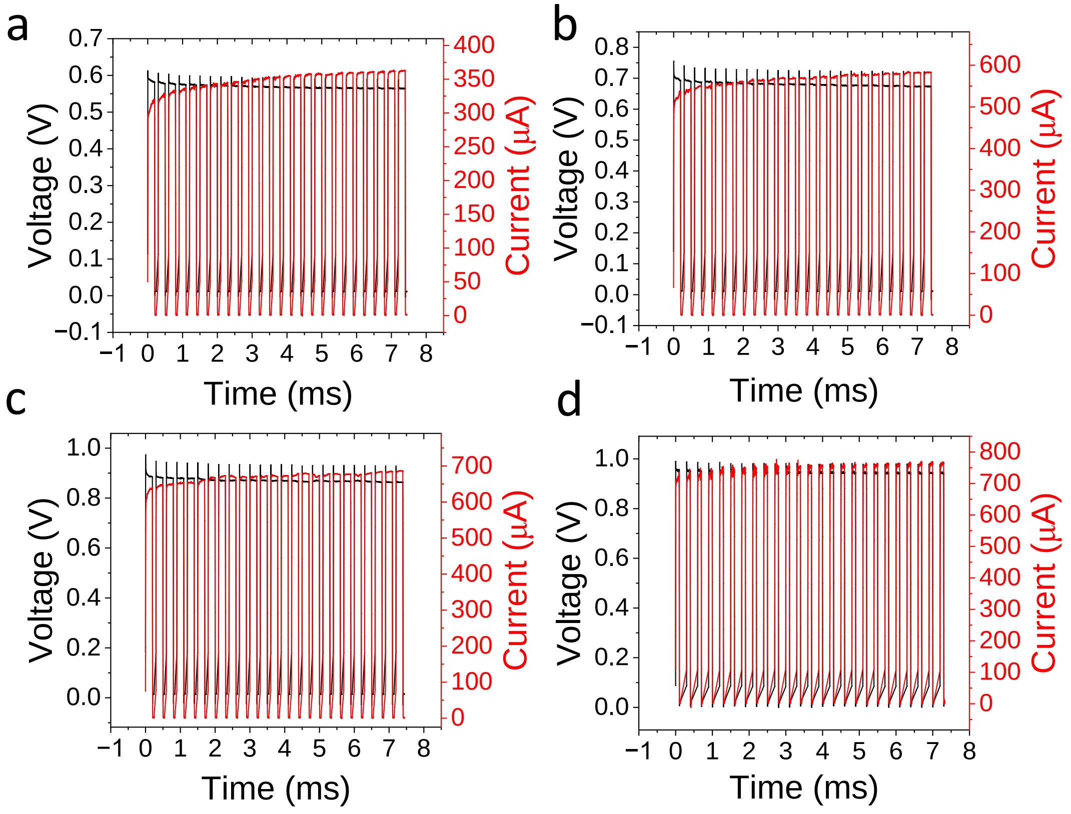

Figure S13. Pulse amplitude modulated synaptic plasticity **(a)** 0.6 V **(b)** 0.7 V **(c)** 0.9V **(d)** 1V.

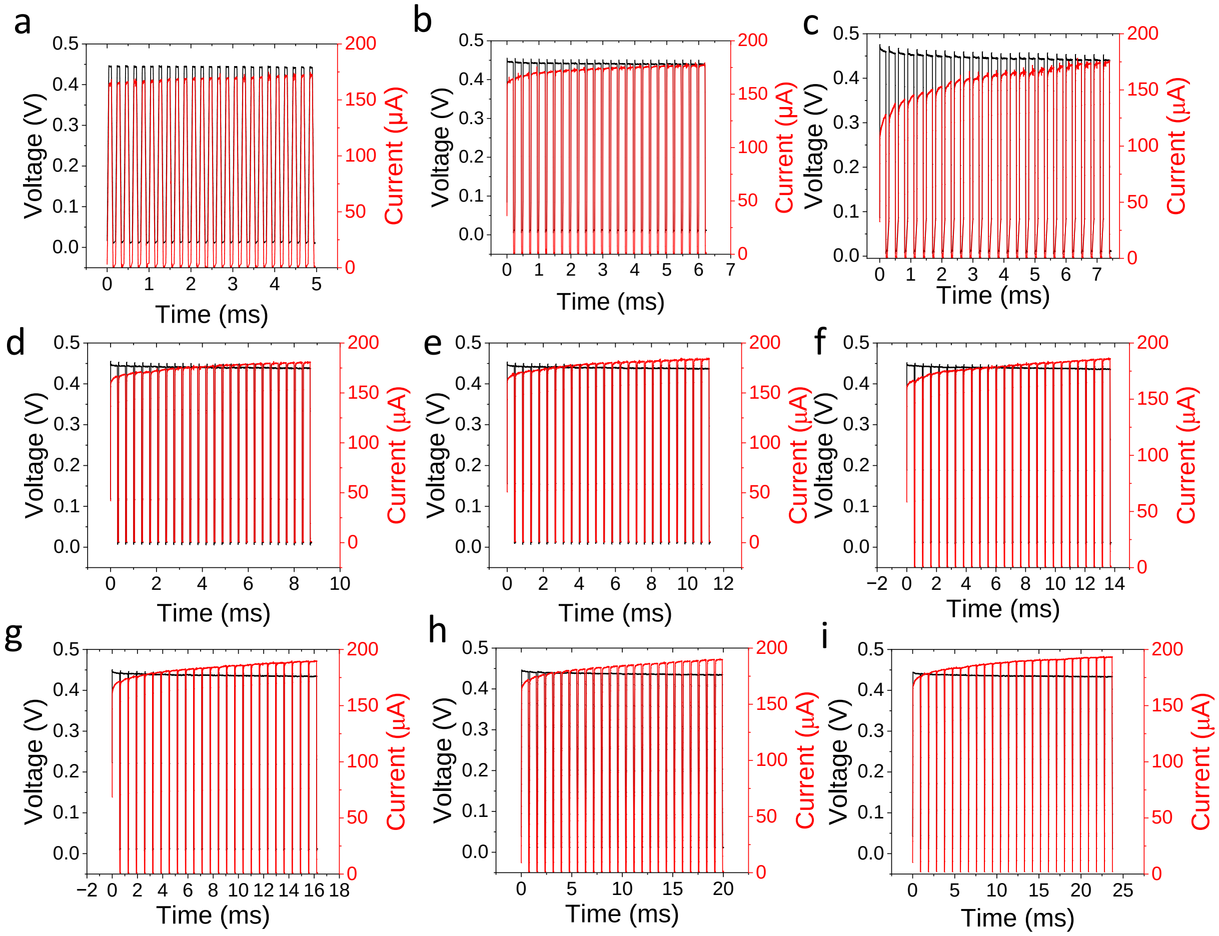

Figure S14. Pulse duration modulated synaptic plasticity **(a)** 0.2 ms **(b)** 0.3 ms **(c)** 0.4 ms **(d)** 0.5 ms **(e)** 0.6 ms **(f)** 0.7 ms **(g)** 0.8 ms **(h)** 0.9 ms **(i)** 1 ms.
